# Prior SARS‐CoV‐2 infection and COVID‐19 vaccine effectiveness against outpatient illness during widespread circulation of SARS‐CoV‐2 Omicron variant, US Flu VE network

**DOI:** 10.1111/irv.13143

**Published:** 2023-05-25

**Authors:** Sara Y. Tartof, Fagen Xie, Ruchi Yadav, Karen J. Wernli, Emily T. Martin, Edward A. Belongia, Manjusha Gaglani, Richard K. Zimmerman, H. Keipp Talbot, Natalie Thornburg, Brendan Flannery, US Flu VE Network Investigators

**Affiliations:** ^1^ Department of Research and Evaluation Kaiser Permanente Southern California Pasadena California USA; ^2^ Department of Health Systems Science Kaiser Permanente Bernard J. Tyson School of Medicine Pasadena California USA; ^3^ Centers for Disease Control and Prevention Atlanta Georgia USA; ^4^ Kaiser Permanente Washington Health Research Institute Seattle Washington USA; ^5^ University of Michigan School of Public Health Ann Arbor Michigan USA; ^6^ Marshfield Clinic Research Institute Marshfield Wisconsin USA; ^7^ Baylor Scott & White Health Temple Texas USA; ^8^ Texas A&M University College of Medicine Temple Texas USA; ^9^ University of Pittsburgh Pittsburgh Pennsylvania USA; ^10^ Vanderbilt University Medical Center Nashville Tennessee USA

**Keywords:** COVID‐19, hybrid immunity, vaccine effectiveness

## Abstract

**Background:**

We estimated combined protection conferred by prior SARS‐CoV‐2 infection and COVID‐19 vaccination against COVID‐19‐associated acute respiratory illness (ARI).

**Methods:**

During SARS‐CoV‐2 Delta (B.1.617.2) and Omicron (B.1.1.529) variant circulation between October 2021 and April 2022, prospectively enrolled adult patients with outpatient ARI had respiratory and filter paper blood specimens collected for SARS‐CoV‐2 molecular testing and serology. Dried blood spots were tested for immunoglobulin‐G antibodies against SARS‐CoV‐2 nucleocapsid (NP) and spike protein receptor binding domain antigen using a validated multiplex bead assay. Evidence of prior SARS‐CoV‐2 infection also included documented or self‐reported laboratory‐confirmed COVID‐19. We used documented COVID‐19 vaccination status to estimate vaccine effectiveness (VE) by multivariable logistic regression by prior infection status.

**Results:**

Four hundred fifty‐five (29%) of 1577 participants tested positive for SARS‐CoV‐2 infection at enrollment; 209 (46%) case‐patients and 637 (57%) test‐negative patients were NP seropositive, had documented previous laboratory‐confirmed COVID‐19, or self‐reported prior infection. Among previously uninfected patients, three‐dose VE was 97% (95% confidence interval [CI], 60%–99%) against Delta, but not statistically significant against Omicron. Among previously infected patients, three‐dose VE was 57% (CI, 20%–76%) against Omicron; VE against Delta could not be estimated.

**Conclusions:**

Three mRNA COVID‐19 vaccine doses provided additional protection against SARS‐CoV‐2 Omicron variant‐associated illness among previously infected participants.

## 1 INTRODUCTION

Recovery from SARS‐CoV‐2 infection is followed by a period of immunologic protection against reinfection. Whether SARS‐CoV‐2 infection confers broad or cross‐reactive protection against new SARS‐CoV‐2 variants is uncertain.^1^, ^2^, ^3^ Vaccination and prior infection are both immunizing events, resulting in robust humoral and cellular immune response.^4^, ^5^, ^6^, ^7^, ^8^ As such, unvaccinated individuals with prior infection could have similar levels of protection as fully vaccinated individuals with no prior infection.^9^, ^10^, ^11^, ^12^, ^13^, ^14^ Further, recent data demonstrate that combined immunity from infection and up to three doses of mRNA vaccine (i.e., hybrid immunity) surpasses that from either vaccination or infection alone.^15^

Accurately identifying prior infections is difficult without prospective seroprevalence studies or laboratory‐confirmed infection. Seroprevalence studies indicate that many SARS‐CoV‐2 infections go undetected.^16^, ^17^ These include asymptomatic infections and those for which testing is not sought or produces false‐negative results. Additionally, COVID‐19 testing access and preferences vary by COVID‐19 immunization status, clinical, and sociodemographic factors, likely contributing to misclassification of prior infection when relying solely on healthcare encounters data.^18^, ^19^ Further, the rapid influx of self‐administered home testing in early 2022 reduced PCR‐based testing at healthcare sites.^20^ Together, these factors contribute to underestimation of prior SARS‐CoV‐2 infection when relying on healthcare encounters data and to preferential clinical testing. Studies that have assessed protection from infection or COVID‐19 vaccine effectiveness (VE) against repeat SARS‐CoV‐2 infection have generally relied on electronic health record (EHR) evidence of a positive laboratory test but have not considered the role of undocumented prior infection. This approach assumes the “unvaccinated” or “unexposed” individuals of the control group to be immunologically naïve. The extent of the impact of this misclassification of previously infected individuals on the evaluation of protection provided by infection against new SARS‐CoV‐2 variants or added benefits of COVID‐19 vaccination has important implications for future prevention strategies.

Previously, we estimated the effectiveness of two or three doses of mRNA COVID‐19 vaccines against SARS‐CoV‐2 Delta and Omicron variant‐associated symptomatic outpatient illness.^21^ Prospective collection of dried blood spots (DBS) from patients provided the opportunity to assess the role of prior SARS‐CoV‐2 infection, including documented or serologic evidence, on VE. Here, we estimated protection conferred by hybrid immunity (prior infection and vaccination) for two‐ and three‐dose mRNA COVID‐19 vaccines via analyses stratified by prior SARS‐CoV‐2 infection.

## 2 METHODS

### 2.1 Study population

This study was conducted by participating institutions in the US Influenza Vaccine Effectiveness (Flu VE) Network with study sites in seven states (CA, MI, PA, TN, TX, WA, and WI) as previously described.^21^ Briefly, active surveillance was conducted at outpatient healthcare facilities or COVID‐19 testing sites for patients aged ≥6 months with acute illness with a symptom duration of ≤10 days. Ill individuals were screened using a standard case definition for COVID‐19‐like illness (CLI) that included at least one of fever, cough, or loss of taste/smell.^22^

Participants completed standardized enrollment questionnaires. Data collected included participant age, gender, race, ethnicity, date of illness onset, symptoms, self‐reported chronic medical condition (heart disease, lung disease, diabetes, cancer, liver/kidney disease, immune suppression, or high blood pressure), the highest level of education, and high‐risk exposure. High‐risk exposures in the 14 days prior to illness were captured with three questions related to healthcare work and close contact with a person with laboratory‐confirmed SARS‐CoV‐2 or CLI (Supplemental Methods).

Participants were asked about their history of positive SARS‐CoV‐2 tests. Participants were asked “Prior to this illness, did you test positive for COVID‐19 (by any test – e.g., rapid, PCR, or antibody test)?” Participants reporting a prior positive test were asked if the most recent positive test was <90 or ≥90 days before the current illness. This activity was reviewed and approved by CDC and each site's Institutional Review Board (see 45 C.F.R. part 46; 21 C.F.R. part 56).

### 2.2 Specimen collection and laboratory testing

Participants had an oropharyngeal and nasal swab specimen collected for SARS‐CoV‐2 molecular testing and finger prick for collection of DBS on filter paper. Participants opting out of finger‐stick blood collection were excluded from this analysis.

Preparation of DBS is described in Supplemental Methods. Briefly, research staff collected whole blood by finger stick and absorbed drops on up to five half‐inch circles on Whatman 903 filter paper cards, which were dried at room temperature, packed with desiccant, and sent to CDC. DBS specimens were tested for immunoglobulin G (IgG) antibodies against three SARS‐CoV‐2 recombinant antigens representing the receptor binding domain (RBD) of the SARS‐CoV‐2 spike‐1 protein, nucleocapsid protein (NP), and an RBD‐NP hybrid antigen using a validated multiplex bead assay (FlexImmArray™ SARS‐CoV‐2 Human IgG Antibody Test, Tetracore, Rockville, MD) on a Luminex MAGPIX instrument with LX200 flow analyzer (Luminex Corporation, Austin, TX). Positive results for the presence of IgG antibodies against SARS‐CoV‐2 NP protein were defined according to the manufacturer's instructions as median fluorescence intensity (MFI) of sample greater than 1.2‐fold the MFI of the human IgG calibrator serum for NP antigen. Samples with equivocal ratios (between ≥0.9 and ≤1.2) were repeated; specimens with final ratios < 0.9‐fold that of NP antigen calibrator serum were defined as anti‐NP IgG seronegative. Anti‐NP IgG seropositivity was considered evidence of previous SARS‐CoV‐2 infection.

### 2.3 Evidence of prior SARS‐CoV‐2 infection

Study sites searched EHR for documented clinical SARS‐CoV‐2 tests since March 2020 for participants and extracted test type and results. Documented prior SARS‐CoV‐2 infection was defined as EHR documentation of positive SARS‐CoV‐2 RT‐PCR or antigen test (including test results imported from outside the healthcare system). *Possible* prior infection was defined as a self‐reported previous positive test for SARS‐CoV‐2 if the most recent positive test had occurred >90 days before the current illness onset. *Confirmed* prior infection was defined as anti‐NP seropositivity or EHR documentation of previous laboratory‐confirmed SARS‐CoV‐2 infection >90 days before the current illness. Confirmed and possible evidence were combined for analyses; sensitivity analysis considered confirmed prior infection only.

### 2.4 COVID‐19 vaccination status

Vaccination receipt was verified in electronic immunization records as previously described.^21^ All vaccine doses during the study period were monovalent mRNA products. Participants were considered vaccinated with two doses if they received two doses of either mRNA vaccine with the second administered ≥14 days before illness onset. We required at least a 16‐day interval between the first and second doses for Pfizer‐BioNTech vaccine and at least a 23‐day interval for Moderna vaccine. If illness onset occurred <7 days after a third dose of mRNA vaccine, the participant was considered to have received two doses.

Participants were considered vaccinated with three doses if they received three mRNA vaccine doses with at least a 16‐day interval between the second and third doses for Pfizer‐BioNTech vaccine and at least a 23‐day interval for Moderna vaccine with the most recent dose received ≥7 days before illness onset.^23^ Those with no EHR‐documented COVID‐19 vaccination before illness onset were considered unvaccinated. Participants who had received one or four doses of mRNA vaccine or any non‐mRNA COVID‐19 vaccine were excluded (Figure S1).

### 2.5 Analyses

We limited all analyses to adults aged ≥18 years for whom a DBS was obtained. Characteristics were compared between those who were SARS‐CoV‐2‐positive at enrollment versus those who tested negative. We determined the distribution of characteristics for those with (1) no evidence of prior SARS‐CoV‐2 infection versus those with confirmed/possible prior infection and (2) prior infection by each data source (NP serology, EHR documentation, self‐report). Descriptive analyses explored the correspondence between RBD seropositivity and COVID‐19 vaccination status among NP‐seronegative participants.

To investigate whether COVID‐19 vaccination provided additional protection beyond that conferred by prior infection, we compared odds of confirmed/possible prior infection among those with SARS‐CoV‐2 positive results at enrollment (i.e., cases) to those who tested negative at enrollment (i.e., controls), stratified by vaccination status (unvaccinated, two doses, three doses). Adjusted odds were estimated using logistic regression models including age, sex, race and ethnicity, site, illness onset week, self‐reported chronic medical condition, and high‐risk SARS‐CoV‐2 exposure.

VE of two or three mRNA COVID‐19 vaccine doses was estimated overall, by time period/variant, and by prior infection status using the test‐negative design. Cases were participants with positive SARS‐CoV‐2 RT‐PCR results at enrollment, and controls were participants with negative SARS‐CoV‐2 results at enrollment. The odds of two‐ or three‐dose mRNA COVID‐19 vaccination among SARS‐CoV‐2‐positive cases versus test‐negative controls were estimated using logistic regression models adjusted for the covariates used in the model above. VE was calculated as (1 − adjusted [or unadjusted] odds ratio) × 100%. VE by the number of doses received was estimated overall and by variant using time periods of predominant Delta (illness onset October 1–December 9, 2021) and Omicron circulation (illness onset December 20, 2021–April 15, 2022). These periods were selected based on the SARS‐CoV‐2 sequencing results from a subset of Flu VE Network SARS‐CoV‐2 cases.^21^ Infections from December 10–19, 2021, were excluded because of variant co‐circulation. Adjusted VE was further stratified by prior infection status (possible/confirmed versus no prior infection).

## 3 RESULTS

From October 1, 2021, to April 15, 2022, 6244 patients aged ≥18 years with CLI were enrolled and tested prospectively for SARS‐CoV‐2 infection using molecular assays. Of these, 1883 provided DBS specimens. Of those, 1577 met the inclusion criteria for this analysis (Table 1). Among these participants, the mean age was 43 years (SD = 16); the majority (1033, 64%) were female, White (1067, 68%), and college educated (970, 62%). Overall, 254 (16%) participants were Hispanic, 442 (28%) reported having a chronic medical condition, and 643 (42%) reported having a high‐risk SARS‐CoV‐2 exposure. Of included participants, 455 (29%) tested SARS‐CoV‐2 positive by RT‐PCR at enrollment, and 1122 (71%) tested negative (Table 1). SARS‐CoV‐2 positivity differed significantly by high‐risk exposure, study site, and week of illness onset. Median days from symptom onset to enrollment and DBS collection was three (range, 0–13; 1 with 11 and 1 with 13).

**TABLE 1:** Participant characteristics by SARS‐CoV‐2 status at enrollment.

	SARS‐CoV‐2 status at enrollment			
	Negative	Positive	All	*P*‐value^a^
Total (row percent)	1122 (71%)	455 (29%)	1577	
Age (years)				0.19
Mean (SD)	42 (16)	43 (15)	43 (16)	
Median (IQR)	40 (30, 56)	41.0 (31, 53)	40.0 (30, 55)	
18–49	741 (66)	314 (69)	1055 (67)	
50–64	247 (22)	101 (22)	348 (22)	
≥65	134 (12)	40 (9)	174 (11)	
Sex				0.15
Female	726 (65)	277 (61)	1003 (64)	
Male	396 (35)	178 (39)	574 (36)	
Race and ethnicity^b^				0.09
White	779 (69)	288 (63)	1067 (68)	
Black	57 (5)	26 (6)	83 (5)	
Hispanic	173 (15)	81 (18)	254 (16)	
Asian	96 (9)	46 (10)	142 (9)	
Other/unknown	17 (2)	14 (3)	31 (2)	
Self‐reported chronic medical condition^c^				0.24
Yes	324 (29)	118 (26)	442 (28)	
No	798 (71)	337 (74)	1135 (72)	
High‐risk SARS‐CoV‐2 exposure^d^				<0.01
Yes	413 (37)	230 (51)	643 (41)	
No	709 (63)	225 (50)	934 (59)	
Highest level of education^e^ obtained				0.52
High school or less	188 (17)	83 (18)	271 (17)	
College	690 (62)	280 (62)	970 (62)	
Advanced degree	240 (21)	92 (20)	332 (21)	
Network site				0.03
California	326 (29)	136 (30)	462 (29)	
Michigan	46 (4)	23 (5)	69 (4)	
Pennsylvania	264 (24)	91 (20)	355 (23)	
Tennessee	144 (13)	40 (9)	184 (12)	
Texas	139 (12)	56 (12)	195 (12)	
Washington	104 (9)	61 (13)	165 (11)	
Wisconsin	99 (9)	48 (11)	147 (9)	
Week of illness onset				<0.01
Oct 10, 2021–Nov 16, 2021	92 (8)	13 (3)	105 (7)	
Nov 17, 2021–Dec 4, 2021	237 (21)	32 (7)	269 (17)	
Dec 5, 2021–Jan 1, 2022	206 (18)	98 (22)	304 (19)	
Jan 2, 2022–Jan 29, 2022	139 (12)	184 (40)	323 (21)	
Jan 30, 2022–Feb 26, 2022	153 (14)	59 (13)	212 (13)	
Feb 27, 2022–Mar 26, 2022	240 (21)	32 (7)	272 (17)	
Mar 27, 2022–Apr 15, 2022	55 (5)	37 (8)	92 (6)	

*Note*: Data reported as number (column percent) unless noted.

^a^ *P*‐value from Chi‐square test comparing COVID‐19 negative with positive.

^b^ Race and Hispanic ethnicity as participant reported at enrollment. Participants could select more than one. “Other” category includes Native Hawaiian or other Pacific Islander and American Indian or Alaska Native.

^c^ Conditions included heart disease, lung disease, diabetes, cancer, liver/kidney disease, immune suppression, or high blood pressure.

^d^ High‐risk SARS‐CoV‐2 exposures included healthcare work in close contact with patients, close contact in the 14 days before illness onset with either a person with laboratory‐confirmed SARS‐CoV‐2 or a household member with laboratory‐confirmed SARS‐CoV‐2 or symptoms consistent with COVID‐19‐like illness.

^e^ Unknown for four SARS‐CoV‐2‐negative participants.

Among 1577 participants, 846 (54%) had evidence of confirmed/possible prior infection (Table 2). EHR sources of prior laboratory‐confirmed SARS‐CoV‐2 infection documented 171 (11%) participants with prior infection. Nearly all prior infections were detected by serologic testing; 790 of 846 (93%) participants had serologic evidence of prior infection. Less than half of prior infections (241/846, 29%) were evident from more than one source. Additionally, 570 (67%) participants only had serologic evidence of prior infection, 14 (2%) only had a documented prior laboratory‐confirmed SARS‐CoV‐2 infection, and 21 (3%) only self‐reported a positive SARS‐CoV‐2 test that was not apparent in EHR. An additional 106 participants self‐reported or had EHR documentation of an infection <90 days prior to an infection at enrollment and were excluded from analyses; 31 (29%) of these participants also had another infection >90 days prior. In all, combining three sources of prior infection data increased the proportion of participants classified as having prior SARS‐CoV‐2 infection by nearly fivefold compared with using EHR documentation alone to define prior infection status.

**TABLE 2:** Characteristics of participants with prior SARS‐CoV‐2 infection category.

						Prior SARS‐CoV‐2 infection status					
	*N*	No evidence of prior infection		Confirmed^a^ ^,^ ^b^ or possible^c^ prior infection		Serologic^a^		Documented^b^		Self‐reported^c^	
	*N*	No.	%	No.	%	No.	%^d^	No.	%^d^	No.	%^d^
	1577	731	46	846	54	790	50	171	11	212	13
Age (years)											
Mean (SD)		42.5 (15.3)		43.1 (15.9)		43.3 (16.0)		43.2 (14.6)		39.3 (14.7)	
18–49	1055	506	48	549	52	507	48	111	11	157	15
50–64	348	150	43	198	57	189	54	45	13	40	11
≥65	174	75	43	99	57	94	54	15	9	15	9
Sex											
Female	1003	458	46	545	54	509	51	125	12	148	15
Male	574	273	48	301	52	281	49	46	8	64	11
Race and ethnicity^e^											
White	1067	482	45	585	55	540	51	115	11	149	14
Black	83	39	47	44	53	44	53	4	5	11	13
Hispanic	254	112	44	142	56	135	53	39	15	37	15
Asian	142	81	57	61	43	57	40	9	6	11	8
Other/unknown	31	17	55	14	45	14	45	4	13	4	13
Highest level of education obtained^f^											
High school or less	271	123	45	148	55	137	51	34	13	40	15
College	970	441	45	529	55	494	51	112	12	151	16
Advanced degree	332	166	50	166	50	156	47	24	7	21	6
Self‐reported chronic medical condition^g^											
Yes	442	213	48	229	52	212	48	45	10	53	12
No	1135	518	46	617	54	578	51	126	11	159	14
High‐risk SARS‐CoV‐2 exposure^h^											
Yes	643	281	44	362	56	340	53	76	12	103	16
No	934	450	48	484	52	450	48	95	10	109	12
Number of COVID‐19 vaccine doses^i^											
Unvaccinated	289	135	47	154	53	137	47	43	15	63	22
2‐dose	647	389	60	258	40	235	36	70	11	81	13
3‐dose	641	207	32	434	68	418	65	58	9	68	11
SARS‐CoV‐2 test result at enrollment											
Positive	455	246	54	209	46	192	42	38	8	41	9
Negative	1122	485	43	637	57	598	53	133	12	171	15

^a^ Nucleocapsid protein (NP) seropositive by multiplex SARS‐CoV‐2 bead assay (FlexImmArray, Tetracore).

^b^ Laboratory confirmed SARS‐CoV‐2 infection documented in electronic health record (EHR) > 90 days before onset of current illness.

^c^ Participant self‐report of prior confirmed COVID‐19 or positive SARS‐CoV‐2 test >90 days before onset of current illness.

^d^ Denominator is the row total for the header row. Categories of prior SARS‐CoV‐2 infection status are not mutually exclusive and sum to more than 100%.

^e^ Race and Hispanic ethnicity as participant reported at enrollment. Participants could select more than one. “Other” category includes Native Hawaiian or other Pacific Islander and American Indian or Alaska Native.

^f^ Unknown for four participants.

^g^ Conditions included heart disease, lung disease, diabetes, cancer, liver/kidney disease, immune suppression, or high blood pressure.

^h^ High‐risk SARS‐CoV‐2 exposures included healthcare work in close contact with patients, close contact in the 14 days before illness onset with either a person with laboratory‐confirmed SARS‐CoV‐2 or a household member with laboratory‐confirmed SARS‐CoV‐2 or symptoms consistent with COVID‐19‐like illness.

^i^ Number of COVID‐19 vaccine doses reported in a participant's electronic health record.

Among participants included in this analysis, 641 (41%) had received three doses and 647 (41%) had received two doses of monovalent mRNA vaccines ≥14 days before illness onset; 289 (18%) were unvaccinated at the time of enrollment. Presence of antibodies to SARS‐CoV‐2 spike protein RBD antigen correlated with EHR‐documented COVID‐19 vaccination status (Table 3). However, 18 of 647 participants with two doses (3%) and nine of 641 participants with three doses (1%) were RBD‐antibody seronegative at enrollment. Of these 27 RBD‐seronegative patients with prior vaccination, the mean and median time since the second dose (*n* = 18) was 240 and 235 days, respectively; the mean and median time since the third dose was 83 and 78 days, respectively (*n* = 18). About half (48%) of the 27 RBD‐seronegative, vaccinated participants had chronic medical conditions, compared with 29% of seropositive, vaccinated participants.

**TABLE 3:** Seroprevalence of antibodies against SARS‐CoV‐2 spike protein Receptor Binding Domain (RBD) by COVID‐19 vaccination status and evidence of prior SARS‐CoV‐2 infection.^a^

	Total^b^	No. (column %) seropositive^a^	No. (column %) seronegative	*P*‐value^c^
Number of COVID‐19 vaccine doses^d^				<0.01
Unvaccinated	267	172 (13)	95 (78)	
2 doses	613	595 (45)	18 (15)	
3 doses	561	552 (42)	9 (7)	
Confirmed or possible prior SARS‐CoV‐2 infection^e^				<0.01
Unvaccinated	148	138 (17)	10 (71)	
2 doses	252	251 (31)	1 (7)	
3 doses	429	426 (52)	3 (21)	
No evidence of prior SARS‐CoV‐2 infection^f^				<0.01
Unvaccinated	119	34 (7)	85 (79)	
2 doses	361	344 (68)	17 (16)	
3 doses	132	126 (25)	6 (6)	

*Note*: RBD, SARS‐CoV‐2 protein receptor binding domain.

^a^ Seropositive for SARS‐CoV‐2 Spike Protein RBD.

^b^ Excludes 136 participants with indeterminate serostatus for RBD (*n* = 7) or NP (*n* = 129) antibody. Twenty‐two participants with indeterminate serostatus were unvaccinated, 34 received two doses of vaccine, and 80 received three doses of vaccine.

^c^ *P*‐value for Chi‐square test comparing proportion seropositive to seronegative.

^d^ Vaccination status based on the number of doses documented in electronic medical record received ≥14 days before illness onset for the second dose or ≥7 days before illness onset for the third dose.

^e^ Includes seropositive nucleocapsid protein (NP) antibody assay, electronic medical record documented positive SARS‐CoV‐2 molecular or antigen test, or participant self‐report of positive SARS‐CoV‐2 positive laboratory test >90 days before onset of current illness.

^f^ Participants NP antibody seronegative without EHR‐documented or self‐reported positive COVID‐19 test >90 days before onset of current illness.

Among 148 unvaccinated participants with confirmed/possible prior SARS‐CoV‐2 infection, 138 (93%) were RBD‐antibody seropositive and NP seronegative, indicating the presence of anti‐Spike protein antibody from prior infection, undocumented vaccination, or both. In contrast, 34 (29%) of 119 unvaccinated participants without confirmed/possible prior SARS‐CoV‐2 infection were RBD‐antibody seropositive and NP seronegative at enrollment, suggesting serologic evidence of prior infection or undocumented vaccination.

### 3.1 Prior SARS‐CoV‐2 infection and risk of SARS‐CoV‐2 at enrollment

The adjusted odds of having SARS‐CoV‐2 at enrollment tended to be reduced for those with prior infection, regardless of vaccination status (Table 4). For the entire enrollment period, prior SARS‐CoV‐2 infection was associated with reduced odds of testing positive for SARS‐CoV‐2 among participants who had received two COVID‐19 mRNA doses (aOR = 0.47; 95% CI, 0.30–0.76) or three doses (aOR = 0.76; 95% CI, 0.49–1.19), but confidence intervals were wide and not statistically significant for three doses. Among two‐dose vaccine recipients during the Omicron‐variant period, the odds of testing positive were significantly reduced among participants with prior infection versus those without prior infection (OR = 0.39, 95% CI, 0.22–0.70). Similarly, the odds of testing positive among three‐dose recipients was 0.81 (95% CI, 0.51–1.29) for those with prior infection versus those without prior infection. Findings were similar when only EHR documentation was used to define prior infection status (Table S1).

**TABLE 4:** Odds ratios for SARS‐CoV‐2 infection among 1577 ill participants with and without evidence^a^ of prior SARS‐CoV‐2 infection by COVID‐19 vaccination status^b^ and SARS‐CoV‐2 virus type.

	Prior infection	Total	No. SARS‐CoV‐2 positive	% positive	Adjusted^c^ OR [95% CI]
Overall					
Unvaccinated	No Prior infection	135	60	44	Referent
	Prior infection	154	45	29	0.59 [0.32, 1.08]
2 doses	No Prior infection	389	129	33	Referent
	Prior infection	258	57	22	0.47 [0.30, 0.76]
3 doses	No Prior infection	207	57	28	Referent
	Prior infection	434	107	25	0.76 [0.49, 1.19]
Delta variant					
Unvaccinated	No Prior infection	43	14	33	Referent
	Prior infection	37	5	14	0.41 [0.11, 1.52]
2 doses	No Prior infection	189	20	11	Referent
	Prior infection	83	8	10	0.84 [0.32, 2.22]
3 doses	No Prior infection	31	1	3	Referent
	Prior infection	33	1	3	NE^d^
Omicron variant					
Unvaccinated	No Prior infection	74	38	51	Referent
	Prior infection	109	38	35	0.86 [0.37, 2.01]
2 doses	No Prior infection	157	93	59	Referent
	Prior infection	158	47	30	0.39 [0.22, 0.70]
3 doses	No Prior infection	162	53	33	Referent
	Prior infection	371	104	28	0.81 [0.51, 1.29]

Abbreviations: CI, confidence interval; NE, not estimates; OR, odds ratio.

^a^ Includes seropositive nucleocapsid protein (NP) antibody assay, electronic medical record documented positive SARS‐CoV‐2 molecular or antigen test, or participant self‐report of positive SARS‐CoV‐2 positive laboratory test >90 days before onset of current illness.

^b^ Vaccination status based on number of doses documented in electronic medical record received ≥14 days before illness onset for the second dose or ≥7 days before illness onset for the third dose.

^c^ Model adjusted for age, sex, race/ethnicity, site, illness onset week, self‐reported chronic medical condition, and high‐risk SARS‐CoV‐2 exposure.

^d^ Adjusted odds ratio not estimated due to too few cases.

### 3.2 Prior SARS‐CoV‐2 infection and COVID‐19 vaccine effectiveness

Among 455 patients who tested SARS‐CoV‐2 positive at enrollment, 186 (41%) had received two COVID‐19 mRNA vaccine doses, 164 (36%) had received three doses, and 209 (46%) had confirmed/possible evidence of prior SARS‐CoV‐2 infection. Among 1122 SARS‐CoV‐2‐negative participants, 461 (41%) and 477 (43%) had received two or three mRNA vaccine doses, respectively, and 637 (57%) had evidence of prior SARS‐CoV‐2 infection.

Overall, adjusted VE for two COVID‐19 vaccine doses among patients with no evidence of prior SARS‐CoV‐2 infection tended to be lower than VE estimates among patients with confirmed/possible prior infection, although neither estimate was statistically significant (Table 5). During the SARS‐CoV‐2 Omicron variant‐predominant period, two‐dose VE was 45% (95% CI, −4 to 70) among those with confirmed/possible prior infection versus −53% (95% CI, −205 to 23) among those without prior infection; three‐dose VE was 57% (95% CI, 20 to 76) versus 23% (95% CI, −72 to 65). Fewer breakthrough infections during the Delta‐variant‐predominant period did not allow for meaningful comparisons between strata. Findings were similar when only EHR documentation was used to define prior infection status (Table S2).

**TABLE 5:** Adjusted^a^ vaccine effectiveness against COVID‐19 by SARS‐CoV‐2 variant stratified by prior infection status.^b^

	Vaccination^c^	Total	SARS‐CoV‐2 positive	% positive	Adjusted^a^ VE [95% CI]
Overall					
No prior infection	Unvaccinated	135	60	44	Referent
	2 doses	389	129	33	17 [−40, 51]
	3 doses	207	57	28	53 [10, 76]
With prior infection	Unvaccinated	154	45	29	Referent
	2 doses	258	57	22	42 [−1, 66]
	3 doses	434	107	25	55 [21, 74]
Delta variant					
No prior infection	Unvaccinated	43	14	33	Referent
	2 doses	189	20	11	70 [11, 90]
	3 doses	31	1	3	NE^d^
With prior infection	Unvaccinated	37	5	14	Referent
	2 doses	83	8	10	23 [−225, 82]
	3 doses	33	1	3	NE^d^
Omicron variant					
No prior infection	Unvaccinated	74	38	51	Referent
	2 doses	157	93	59	−53 [−205, 23]
	3 doses	162	53	33	23 [−72, 65]
With prior infection	Unvaccinated	109	38	35	Referent
	2 doses	158	47	30	45 [−4, 70]
	3 doses	371	104	28	57 [20, 76]

Abbreviations: CI, confidence interval; NE, not estimates; VE, vaccine effectiveness.

^a^ Models adjusted for age, sex, race/ethnicity, site, illness onset week, self‐reported chronic medical condition, and high‐risk SARS‐CoV‐2 exposure.

^b^ Includes seropositive nucleocapsid protein (NP) antibody assay, electronic medical record documented positive SARS‐CoV‐2 molecular or antigen test, or participant self‐report of positive SARS‐CoV‐2 positive laboratory test >90 days before onset of current illness.

^c^ Vaccination status based on number of doses documented in electronic medical record received ≥14 days before illness onset for the second dose or ≥7 days before illness onset for the third dose.

^d^ Adjusted vaccine effectiveness not estimated due to too few cases.

## 4 DISCUSSION

Understanding the magnitude of protection from infection and the potential additional protection from COVID‐19 vaccination (i.e., “hybrid immunity”) against illness associated with new SARS‐CoV‐2 variant viruses is important for future COVID‐19 vaccine programs. Here, we found that confirmed or possible prior infection was associated with protection against current SARS‐CoV‐2 infection, regardless of vaccination status during the early 2022 Omicron‐variant wave. Vaccination‐conferred immunity among patients with no evidence of prior SARS‐CoV‐2 infection appeared to vary by variant, where two and three doses of COVID‐19 mRNA vaccines provided statistically significant protection against SARS‐CoV‐2‐associated illness during the Delta variant‐predominant period, but no statistically significant protection during the Omicron‐variant period.

For patients with evidence of prior infection, three doses of COVID‐19 mRNA monovalent vaccines provided additional protection against illness during the Omicron‐variant period in the adjusted model, but we could not measure statistically significant additional protection from two vaccine doses or among previously uninfected participants who received two or three vaccine doses. Cases with prior infection were insufficient in the Delta period to assess the role of vaccine during that time. Protection from primary‐series vaccination is short‐lived against Omicron infections,^24^, ^25^ and vaccination with monovalent formulations of mRNA vaccines have been demonstrated to confer weaker protection against Omicron compared with Delta.^26^ These findings are also consistent with work demonstrating that a third dose of BNT162b2 vaccine was needed for induction of consistent neutralizing antibody titers against either BA.1 or BA.2.^27^, ^28^ Relatively weak naturally acquired protective immunity against Omicron in our findings is consistent with waning over time and/or immune evasion of new variants in the absence of boosting with updated vaccines.

A matched test‐negative study from Qatar also found that three vaccine doses and EHR‐confirmed previous infection conferred the greatest protection against SARS‐CoV‐2 Omicron‐variant infection, compared with two doses and prior EHR‐confirmed infection and three doses without EHR‐confirmed prior infection.^15^ However, those with two doses of vaccine and previous infection were afforded similar levels of protection as those with three doses and no previous infection, which was similar to protection from previous infection alone. In our study among persons with no evidence of prior infection, the effectiveness of three doses was similar to that of two doses among previously infected persons. Further, this study was conducted during a period when the US‐based population had high vaccination coverage or prior infection, which may have lessened our ability to discriminate between protection from vaccine or prior infection.

Most persons with SARS‐CoV‐2 infection generate detectable anti‐SARS‐CoV‐2 antibodies, with studies reporting seroconversion rates >90%.^29^, ^30^ The consistency in antibody response allows for highly sensitive serologically based detection methods for prior infections. Here, we found that EHR determination of prior infection alone was not very informative; supplementing those data with NP serology resulted in a fivefold increase in evidence of prior infection. Although EHR‐documented evidence of prior infection is highly specific, it is not sensitive and likely misses a large proportion of those not tested, tested outside the home healthcare system, or self‐tested with antigen‐based kits.^20^ Yet, most large‐scale VE studies to date define prior infection by EHR data sources alone. Although NP serology has higher sensitivity and specificity to estimate prior infection than other modes of ascertainment, future data regarding sensitivity and specificity by time since infection are needed.

RBD serology findings can also inform our understanding of serologic test performance. Seropositivity for SARS‐CoV‐2 spike protein RBD among unvaccinated patients likely indicates prior infection. However, in this study, we identified 34 patients with RBD seropositivity who were seronegative for NP antibodies and were also unvaccinated, suggesting that some prior infections, including potentially asymptomatic infections, may have been missed because of waning of anti‐NP antibody, which occurs more quickly than RBD antibodies.^31^ It is also possible that these patients had prior COVID‐19 vaccination events that were not captured in the EHR. Conversely, not all vaccinated people were RBD seropositive, where 27 individuals were RBD‐seronegative but had documentation of two or three doses of mRNA vaccine. As with NP serology, further studies are needed on infection‐ and vaccination‐elicited RBD antibody durability and performance as a biomarker in epidemiologic research.

There are limitations to this study. For NP serology findings, timing of infection and infecting variant are unknown; we required that self‐reported and EHR‐documented infections occurred ≥90 days prior to illness onset, but we were not able to gauge time of infection using serologic evidence. It is possible that an infection detected at the time of enrollment could have been a prolonged acute respiratory illness rather than a “previous infection” as defined by NP serology. Some vaccinated persons may not seroconvert for NP after infection,^32^ which could lead to misclassification of prior infection in a subset of participants. Our sample size was limited, and some estimates were imprecise, which constrained our ability to compare trends in hybrid immunity over time and variant. Further, variant‐specific analyses were defined by secular period rather than sequencing, which may result in some misclassification. We were not able to differentiate BA.1 versus BA.2. However, studies have demonstrated that neutralizing antibody titers against BA.1 and BA.2 were similar and that robust neutralizing antibody titers against BA.2 developed in those previously infected with BA.1, which suggests a substantial degree of cross‐reactive immunity.^27^ We only capture those that sought medical care or testing in an outpatient healthcare setting and were willing to participate and provide a DBS. Although those that seek RT‐PCR testing may differ from those who test at home or do not test, the test‐negative design equalizes healthcare‐seeking behavior between our comparison groups and minimizes potential bias by vaccination status. Participants who did not provide a DBS specimen were similar to those who did with respect to vaccination status (data not shown). Finally, our analyses are based on the monovalent mRNA COVID‐19 vaccine formulations and may not correspond to findings derived from bivalent formulations.

An improved understanding of cross‐protection elicited by infection and vaccination is needed to inform future vaccine formulations and vaccination recommendations. The optimal vaccination strategy for previously infected individuals would boost protective immunity from natural infection. If natural immunity fosters cross‐protection against emerging variants, formulations that include more cross‐reactive antigens may be necessary to improve VE. As new variants continue to emerge, ongoing analyses of cross‐protection between strains will be important to inform vaccine programs.

## AUTHOR CONTRIBUTIONS

**Sara Y. Tartof:** Conceptualization; data curation; formal analysis; funding acquisition; investigation; methodology; project administration; resources; supervision; writing—original draft; writing—review and editing. **Fagen Xie:** Formal analysis; writing—review and editing. **Ruchi Yadav:** Conceptualization; data curation; formal analysis; investigation; methodology; writing—review and editing. **Karen J. Wernli:** Data curation; funding acquisition; investigation; methodology; project administration; resources; supervision; writing—review and editing. **Emily T. Martin:** Conceptualization; data curation; funding acquisition; investigation; methodology; project administration; resources; supervision; writing—review and editing. **Edward A. Belongia:** Conceptualization; data curation; funding acquisition; investigation; methodology; project administration; resources; supervision; writing—review and editing. **Manjusha Gaglani:** Conceptualization; data curation; funding acquisition; investigation; methodology; project administration; resources; supervision; writing—review and editing. **Richard K. Zimmerman:** Conceptualization; data curation; funding acquisition; investigation; methodology; project administration; resources; supervision; writing—review and editing. **H. Keipp Talbot:** Conceptualization; data curation; funding acquisition; investigation; methodology; project administration; resources; supervision; writing—review and editing. **Natalie Thornburg:** Conceptualization; data curation; investigation; methodology; writing—review and editing. **Brendan Flannery:** Conceptualization; funding acquisition; investigation; methodology; project administration; resources; supervision; writing—review and editing.

## CONFLICT OF INTEREST STATEMENT

All authors completed an ICJME form for disclosure of potential conflicts of interest. Dr. Gaglani reports grants from CDC‐Abt, CDC‐Vanderbilt, and CDC‐Westat. Dr. Martin reports grants from Merck. Dr. Zimmerman reports grants from Sanofi Pasteur. All other authors report no potential conflicts of interest.

### PEER REVIEW

The peer review history for this article is available at https://www.webofscience.com/api/gateway/wos/peer-review/10.1111/irv.13143.

## ETHICS APPROVAL STATEMENT

This activity was reviewed and approved by CDC and each site's Institutional Review Board [see 45 C.F.R. part 46; 21 C.F.R. part 56].

## DISCLOSURE

The findings and conclusions are those of the authors and do not necessarily represent the official position of the Centers for Disease Control and Prevention.

## Supporting information


**Data S1.** **Supplemental Methods.** Enrollment survey questions related to COVID‐19; FlexImmArray SARS‐CoV‐2 IgG Assay kit

**Table S1.** Odds Ratios for SARS‐CoV‐2 infection among ill participants with and without evidence of prior SARS‐CoV‐2 infection by COVID‐19 vaccination status and SARS‐CoV‐2 virus type.
**Table S2.** Adjusted vaccine effectiveness against COVID‐19 by SARS‐CoV‐2 variant stratified by prior infection status

**Figure S1.** Study flow diagram

## Data Availability

Data may be made available by written request to the corresponding author.

## References

[1] Altarawneh HN , Chemaitelly H , Hasan MR , et al. Protection against the Omicron variant from previous SARS‐CoV‐2 infection. N Engl J Med. 2022;386(13):1288‐1290. doi:10.1056/NEJMc2200133 35139269 PMC8849180

[2] Altarawneh HN , Chemaitelly H , Ayoub HH , et al. Protection of SARS‐CoV‐2 natural infection against reinfection with the Omicron BA.4 or BA.5 subvariants. medRxiv 2022: 2022.07.11.22277448.

[3] Malato J , Ribeiro RM , Fernandes E , et al. Rapid waning of protection induced by prior BA.1/BA.2 infection against BA.5 infection. medRxiv 2022: 2022.08.16.22278820.

[4] Cromer D , Juno JA , Khoury D , et al. Prospects for durable immune control of SARS‐CoV‐2 and prevention of reinfection. Nat Rev Immunol. 2021;21(6):395‐404. doi:10.1038/s41577-021-00550-x 33927374 PMC8082486

[5] Grigoryan L , Pulendran B . The immunology of SARS‐CoV‐2 infections and vaccines. Semin Immunol. 2020;50:101422. doi:10.1016/j.smim.2020.101422 33262067 PMC7670910

[6] Post N , Eddy D , Huntley C , et al. Antibody response to SARS‐CoV‐2 infection in humans: a systematic review. PLoS ONE. 2020;15(12):e0244126. doi:10.1371/journal.pone.0244126 33382764 PMC7775097

[7] Shrotri M , van Schalkwyk MCI , Post N , et al. T cell response to SARS‐CoV‐2 infection in humans: a systematic review. PLoS ONE. 2021;16(1):e0245532. doi:10.1371/journal.pone.0245532 33493185 PMC7833159

[8] Wang Z , Schmidt F , Weisblum Y , et al. mRNA vaccine‐elicited antibodies to SARS‐CoV‐2 and circulating variants. Nature. 2021;592(7855):616‐622. doi:10.1038/s41586-021-03324-6 33567448 PMC8503938

[9] Goel RR , Apostolidis SA , Painter MM , et al. Distinct antibody and memory B cell responses in SARS‐CoV‐2 naive and recovered individuals following mRNA vaccination. Sci Immunol. 2021;6(58). doi:10.1126/sciimmunol.abi6950 PMC8158969 33858945

[10] Shenai MB , Rahme R , Noorchashm H . Equivalency of protection from natural immunity in COVID‐19 recovered versus fully vaccinated persons: a systematic review and pooled analysis. Cureus. 2021;13(10):e19102. doi:10.7759/cureus.19102 34868754 PMC8627252

[11] Bozio CH , Grannis SJ , Naleway AL , et al. Laboratory‐confirmed COVID‐19 among adults hospitalized with COVID‐19‐like illness with infection‐induced or mRNA vaccine‐induced SARS‐CoV‐2 immunity—nine states, January‐September 2021. MMWR Morb Mortal Wkly Rep. 2021;70(44):1539‐1544. doi:10.15585/mmwr.mm7044e1 34735425 PMC8568091

[12] Ebinger JE , Fert‐Bober J , Printsev I , et al. Antibody responses to the BNT162b2 mRNA vaccine in individuals previously infected with SARS‐CoV‐2. Nat Med. 2021;27(6):981‐984. doi:10.1038/s41591-021-01325-6 33795870 PMC8205849

[13] Gobbi F , Buonfrate D , Moro L , et al. Antibody response to the BNT162b2 mRNA COVID‐19 vaccine in subjects with prior SARS‐CoV‐2 infection. Viruses. 2021;13(3):422. doi:10.3390/v13030422 33807957 PMC8001674

[14] Krammer F , Srivastava K , Alshammary H , et al. Antibody responses in seropositive persons after a single dose of SARS‐CoV‐2 mRNA vaccine. N Engl J Med. 2021;384(14):1372‐1374. doi:10.1056/NEJMc2101667 33691060 PMC8008743

[15] Altarawneh HN , Chemaitelly H , Ayoub HH , et al. Effects of previous infection and vaccination on symptomatic Omicron infections. N Engl J Med. 2022;387(1):21‐34. doi:10.1056/NEJMoa2203965 35704396 PMC9258753

[16] Kalish H , Klumpp‐Thomas C , Hunsberger S , et al. Undiagnosed SARS‐CoV‐2 seropositivity during the first 6 months of the COVID‐19 pandemic in the United States. Sci Transl Med. 2021;13(601). doi:10.1126/scitranslmed.abh3826 PMC8432952 34158410

[17] Li R , Pei S , Chen B , et al. Substantial undocumented infection facilitates the rapid dissemination of novel coronavirus (SARS‐CoV‐2). Science. 2020;368(6490):489‐493. doi:10.1126/science.abb3221 32179701 PMC7164387

[18] Rentsch CT , Kidwai‐Khan F , Tate JP , et al. Patterns of COVID‐19 testing and mortality by race and ethnicity among United States veterans: a nationwide cohort study. PLoS Med. 2020;17(9):e1003379. doi:10.1371/journal.pmed.1003379 32960880 PMC7508372

[19] Salomon JA , Reinhart A , Bilinski A , et al. The US COVID‐19 trends and impact survey: continuous real‐time measurement of COVID‐19 symptoms, risks, protective behaviors, testing, and vaccination. Proc Natl Acad Sci U S a. 2021;118(51). doi:10.1073/pnas.2111454118 PMC8713763 34903656

[20] Rader B , Gertz A , Iuliano AD , et al. Use of at‐home COVID‐19 tests—United States, August 23, 2021‐March 12, 2022. MMWR Morb Mortal Wkly Rep. 2022;71(13):489‐494. doi:10.15585/mmwr.mm7113e1 35358168 PMC8979595

[21] Kim S , Chung JR , Talbot HK , et al. Effectiveness of two and three mRNA COVID‐19 vaccine doses against Omicron‐ and Delta‐related outpatient illness among adults, October 2021–February 2022. Influenza Other Respi Viruses. 2022;1‐11. 10.1111/irv.13029 PMC9353375 36825251

[22] Chung JR , Kim SS , Jackson ML , et al. Clinical symptoms among ambulatory patients tested for SARS‐CoV‐2. Open forum. Infect Dis. 2021;8(1):ofaa576. doi:10.1093/ofid/ofaa576 PMC7717425 33537361

[23] Tenforde MW , Patel MM , Gaglani M , et al. Effectiveness of a third dose of Pfizer‐BioNTech and Moderna vaccines in preventing COVID‐19 hospitalization among immunocompetent and immunocompromised adults—United States, August‐December 2021. MMWR Morb Mortal Wkly Rep. 2022;71(4):118‐124. doi:10.15585/mmwr.mm7104a2 35085218 PMC9351530

[24] Andrews N , Stowe J , Kirsebom F , et al. Covid‐19 vaccine effectiveness against the Omicron (B.1.1.529) variant. N Engl J Med. 2022;386(16):1532‐1546. doi:10.1056/NEJMoa2119451 35249272 PMC8908811

[25] Chemaitelly H , Ayoub HH , AlMukdad S , et al. Duration of mRNA vaccine protection against SARS‐CoV‐2 Omicron BA.1 and BA.2 subvariants in Qatar. Nat Commun. 2022;13(1):3082. doi:10.1038/s41467-022-30895-3 35654888 PMC9163167

[26] Tartof SY , Slezak JM , Puzniak L , et al. Immunocompromise and durability of BNT162b2 vaccine against severe outcomes due to Omicron and Delta variants. Lancet Respir Med. 2022;10(7):e61‐e62. doi:10.1016/S2213-2600(22)00170-9 35533699 PMC9075856

[27] Yu J , Collier AY , Rowe M , et al. Neutralization of the SARS‐CoV‐2 Omicron BA.1 and BA.2 variants. N Engl J Med. 2022;386(16):1579‐1580. doi:10.1056/NEJMc2201849 35294809 PMC9006770

[28] Liu L , Iketani S , Guo Y , et al. Striking antibody evasion manifested by the Omicron variant of SARS‐CoV‐2. Nature. 2022;602(7898):676‐681. doi:10.1038/s41586-021-04388-0 35016198

[29] Gudbjartsson DF , Norddahl GL , Melsted P , et al. Humoral immune response to SARS‐CoV‐2 in Iceland. N Engl J Med. 2020;383(18):1724‐1734. doi:10.1056/NEJMoa2026116 32871063 PMC7494247

[30] Wang H , Yuan Y , Xiao M , et al. Dynamics of the SARS‐CoV‐2 antibody response up to 10 months after infection. Cell Mol Immunol. 2021;18(7):1832‐1834. doi:10.1038/s41423-021-00708-6 34099890 PMC8182358

[31] Ortega N , Ribes M , Vidal M , et al. Seven‐month kinetics of SARS‐CoV‐2 antibodies and role of pre‐existing antibodies to human coronaviruses. Nat Commun. 2021;12(1):4740. doi:10.1038/s41467-021-24979-9 34362897 PMC8346582

[32] Follmann D , Janes HE , Buhule OD , et al. Antinucleocapsid antibodies after SARS‐CoV‐2 infection in the blinded phase of the randomized, placebo‐controlled mRNA‐1273 COVID‐19 Vaccine Efficacy linical trial. Ann Intern Med. 2022;175(9):1258‐1265. doi:10.7326/M22-1300 35785530 PMC9258784

