# Supplementary material for: Prior SARS‐CoV‐2 infection and COVID‐19 vaccine effectiveness against outpatient illness during widespread circulation of SARS‐CoV‐2 Omicron variant, US Flu VE network
Source: Influenza Other Respir Viruses. 2023 May 25;17(5):e13143. doi: 10.1111/irv.13143 (PMC10209645; doi:10.1111/irv.13143)
Supplement: Supplementary file 1 — Data S1. Supplemental Methods. Enrollment survey questions related to COVID‐19; FlexImmArray SARS‐CoV‐2 IgG Assay kit [file IRV-17-e13143-s003.docx]

**Supplemental Methods**

*Enrollment survey questions related to COVID-19:*

1. Have you taken one or more rapid COVID-19 tests at home for this illness?

[Yes, No, Don’t know, Refused to answer]

1a. [If yes to 1] Was any rapid at-home test positive?

[Yes, No, Don’t know, Refused to answer]

1b. [If yes to 1a] When was your first positive (exact or best estimate)?

[MMDDYY, Don’t know, Refused to answer]

1. Did you work in a healthcare setting and have close contact with patients during the 14 days before your illness began? Close contact means being within 6 feet of a patient.

[Yes, No, Don’t know, Refused to answer]

1. Did you have contact with a lab-confirmed COVID-19 case in the 14 days before your symptoms started?

[Yes, No, Don’t know, Refused to answer]

1. During the 14 days before you became ill, did anyone else in the household have a positive test for COVID-19 or a respiratory illness with cough, fever, chills, or loss of taste or smell?

[Yes, No, Don’t know, Refused to answer]

1. *Prior* to this illness, did you test positive for the novel coronavirus, or COVID-19 (by any test – for example rapid test, PCR test, or antibody test)?

[Yes, No, Don’t know, Refused to answer]

5a. [If yes to 5] Was the positive test

[In the last 3 months (90 days from today, More than 3 months (90 days) ago, Don’t know, Refused to answer]

*FlexImmArray SARS-CoV-2 IgG Assay kit:*

This assay utilizes magnetic microspheres coupled with unique recombinant proteins specific for SARS-CoV-2 (RBD, NP, and RBD/NP fusion). The assay also includes four different internal controls for monitoring each step of assay performance. External positive control, negative control, and calibrator reagents are also provided in the kit and run-in duplicate on every assay plate. Extracted samples from dry blood spots tested in duplicate were diluted 1:400 in assay kit sample dilution buffer, immediately mixed with the antigen-coated microspheres in a 96-well plate and incubated for 20 minutes by gentle shaking at room temperature protected from light. Plates were washed four times with assay wash buffer, and DBS elute IgG antibodies were detected using anti-human IgG conjugated to phycoerythrin by incubation at 20 min under gentle shaking protected from light. The microspheres resuspended in wash buffer were analyzed using a Luminex MAGPIX instrument and a Luminex LX 200 flow analyzer (Luminex Corporation, Austin, TX) with a target of 50 beads per region. The assay cutoff established by the manufacturer were used for scoring test positive samples and any indeterminate (equivocal) samples were repeated as recommended by the test manufacturer.
