# Supplementary material for: Prior SARS‐CoV‐2 infection and COVID‐19 vaccine effectiveness against outpatient illness during widespread circulation of SARS‐CoV‐2 Omicron variant, US Flu VE network
Source: Influenza Other Respir Viruses. 2023 May 25;17(5):e13143. doi: 10.1111/irv.13143 (PMC10209645; doi:10.1111/irv.13143)
Supplement: Supplementary file 2 — Table S1. Odds Ratios for SARS‐CoV‐2 infection among ill participants with and without evidence of prior SARS‐CoV‐2 infection by COVID‐19 vaccination status and SARS‐CoV‐2 virus type. Table S2. Adjusted vaccine effectiveness against COVID‐19 by SARS‐CoV‐2 variant stratified by prior infection status [file IRV-17-e13143-s001.docx]

**Supplemental Table 1.** Odds Ratios for SARS-CoV-2 infection among ill participants with and without evidence^1^ of prior SARS-CoV-2 infection by COVID-19 vaccination status^2^ and SARS-CoV-2 virus type.

|  | **Prior infection** | **Total** | **SARS-CoV-2 positive** | **% positive** | **Adjusted^3^ OR [95% CI]** |
| --- | --- | --- | --- | --- | --- |
| **Overall** |  |  |  |  |  |
| Unvaccinated | No Prior infection | 246 | 92 | 37 | Referent |
|  | Prior infection | 43 | 13 | 30 | 0.67 [0.27, 1.65] |
| 2 doses | No Prior infection | 577 | 174 | 30 | Referent |
|  | Prior infection | 70 | 12 | 17 | 0.45 [0.21, 0.95] |
| 3 doses | No Prior infection | 583 | 151 | 26 | Referent |
|  | Prior infection | 58 | 13 | 22 | 0.68 [0.31, 1.47] |
| **Omicron variant** |  |  |  |  |  |
| Unvaccinated | No Prior infection | 146 | 63 | 43 | Referent |
|  | Prior infection | 37 | 13 | 35 | 0.94 [0.32, 2.70] |
| 2 doses | No Prior infection | 275 | 128 | 47 | Referent |
|  | Prior infection | 40 | 12 | 30 | 0.54 [0.23, 1.28] |
| 3 doses | No Prior infection | 479 | 144 | 30 | Referent |
|  | Prior infection | 54 | 13 | 24 | 0.70 [0.32, 1.52] |

CI, confidence interval; OR, odds ratio

^1^ Only includes electronic medical record documented positive SARS-CoV-2 molecular or antigen test >90 days before onset of current illness.

^2^ Vaccination status based on number of doses documented in electronic medical record received ≥14 days before illness onset for 2^nd^ dose or ≥7 days before illness onset for 3^rd^ dose.

^3^ Model adjusted for age, sex, race/ethnicity, site, illness onset week, self-reported chronic medical condition, high-risk SARS-CoV-2 exposure.

**Supplemental Table 2.** Adjusted^1^ vaccine effectiveness against COVID-19 by SARS-CoV-2 variant stratified by prior infection status^2^

|  | **Vaccination^3^** | **Total** | **SARS-CoV-2 positive** | **% positive** | **Adjusted^1^ VE [95% CI]** |
| --- | --- | --- | --- | --- | --- |
| **Overall** |  |  |  |  |  |
| No prior infection | Unvaccinated | 246 | 92 | 37 | Referent |
|  | 2 doses | 577 | 174 | 30 | 22 [-13, 47] |
|  | 3 doses | 583 | 151 | 26 | 57 [35, 72] |
| With prior infection | Unvaccinated | 43 | 13 | 30 | Referent |
|  | 2 doses | 70 | 12 | 17 | 76 [10, 94] |
|  | 3 doses | 58 | 13 | 22 | 80 [2, 96] |
| **Omicron variant** |  |  |  |  |  |
| No prior infection | Unvaccinated | 146 | 63 | 43 | Referent |
|  | 2 doses | 275 | 128 | 47 | -5 [-68, 34] |
|  | 3 doses | 479 | 144 | 30 | 44 [-8, 66] |
| With prior infection | Unvaccinated | 37 | 13 | 35 | Referent |
|  | 2 doses | 40 | 12 | 30 | 68 [-23, 92] |
|  | 3 doses | 54 | 13 | 24 | 75 [-35, 95] |

CI, confidence interval; VE, vaccine effectiveness

^1^ Models adjusted for age, sex, race/ethnicity, site, illness onset week, self-reported chronic medical condition, high-risk SARS-CoV-2 exposure.

^2^ Only includes electronic medical record documented positive SARS-CoV-2 molecular or antigen test >90 days before onset of current illness.

^3^ Vaccination status based on number of doses documented in electronic medical record received ≥14 days before illness onset for 2^nd^ dose or ≥7 days before illness onset for 3^rd^ dose.
