# Supplementary figures and images for: Prior SARS‐CoV‐2 infection and COVID‐19 vaccine effectiveness against outpatient illness during widespread circulation of SARS‐CoV‐2 Omicron variant, US Flu VE network
Source: Influenza Other Respir Viruses. 2023 May 25;17(5):e13143. doi: 10.1111/irv.13143 (PMC10209645; doi:10.1111/irv.13143)

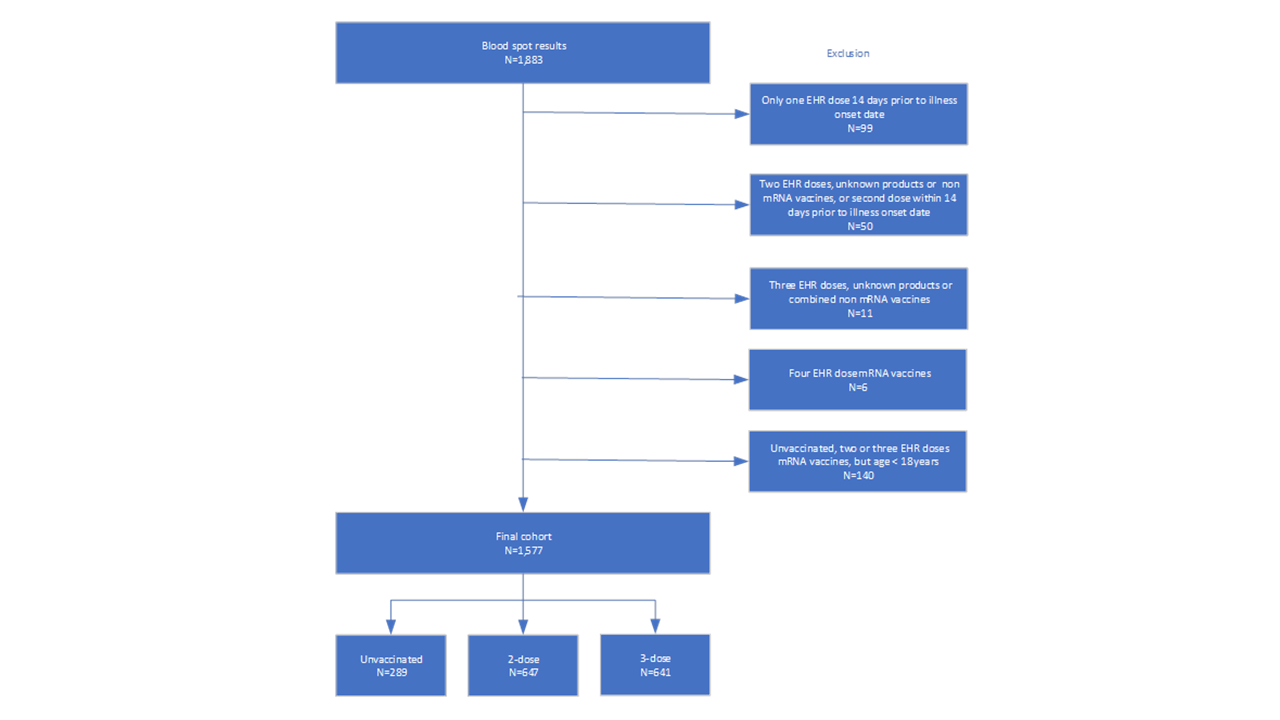

Supplement: Supplementary file 3 — Figure S1. Study flow diagram [file IRV-17-e13143-s002.tif]
